# Loss of PLZF Expression in Prostate Cancer by Immunohistochemistry Correlates with Tumor Aggressiveness and Metastasis

**DOI:** 10.1371/journal.pone.0121318

**Published:** 2015-03-25

**Authors:** Guang-Qian Xiao, Pamela Unger, Qi Yang, Yayoi Kinoshita, Kyra Singh, Loralee McMahon, Kent Nastiuk, Kai Sha, John Krolewski, David Burstein

**Affiliations:** 1 Departments of Pathology, University of Rochester Medical Center, Rochester, New York, United States of America; 2 Departments of Pathology, Mount Sinai Medical Center, New York, New York, United States of America; 3 Departments of Pathology, Lenox Hill Hospital, New York, New York, United States of America; University of Kentucky College of Medicine, UNITED STATES

## Abstract

PLZF is a transcription repressor, which plays a critical role in development, spermatogenesis and oncogenesis. Down-regulation of PLZF has been found in various tumor cell lines. There has been virtually no tissue study on the expression of PLZF in prostate cancer (PCa). PCa is a heterogeneous disease, most of which are indolent and non-lethal. Currently there are no biomarkers that distinguish indolent from aggressive PCa; therefore there is an urgent need for such markers to provide clinical decision support. This study aimed to investigate the expression of PLZF by immunohistochemistry in different grade as well as metastatic PCa and to correlate the alteration of PLZF expression with PCa aggressiveness. We studied a total of 83 primary PCa from biopsies, 43 metastatic PCa and 8 paired primary and metastatic PCa from radical prostatectomies with lymph node dissection. Our results demonstrated that PLZF was strongly expressed in almost all (~100%) benign luminal cells (n=77) and low grade (Gleason pattern 3) PCa (n=70) and weak or absent (100%) in basal cells (n=70). Decreased or lost expression of PLZF was evidenced in 26% of high-grade (Gleason 4 and 5) primary PCa (n=70) and 84% metastatic PCa (n=43). The primary high grade PCa in the prostatectomies shared similar PLZF loss/decrease and histomorphology to that of paired parallel lymph node metastases. These data demonstrated that down-regulation of PLZF is an important molecular process for tumor progression and loss of PLZF expression detected by routine immunohistochemistry is a promising and valuable biomarker for PCa aggressiveness and metastasis in the personalized care of PCa.

## Introduction

Prostate cancer (PCa) is the most common cancer among men in the United States [1]. With the increasing public awareness of PCa, the widespread use of prostate-specific antigen (PSA) serum levels as a screening modality, and trans-rectal ultrasonography to target specific lesions, prostatic needle core biopsies have resulted in increased clinical detection of cancer. PCa is a heterogenous disease, the majority of which have an indolent behavior [2,3]. Despite improvements in early detection, there are currently no reliable biomarkers to effectively distinguish men with high risk disease from the indolent majority and it has been argued for decades that a large number of PCa might have been overtreated [2,4,5]. Therefore, there is an urgent need for such a biomarker that can be used to identify aggressive PCa and start early curative treatment.

Promyelocytic leukemia zinc finger protein (PLZF), also known as Zbtb16 or Zfp145, first identified in a patient with acute promyelocytic leukemia, is a zinc finger transcription factor belonging to the POZ-Krüppel (POK) family that binds to specific DNA sequences with its carboxy-terminal zinc fingers and suppresses transcription by recruiting co-repressors with its aminoterminal POZ domain [6,7]. Functioning in the nucleus, PLZF affects diverse signaling including cell cycle, differentiation, and programmed cell death in hematopoietic cells [7] as well as, more recently, solid tumors [8–10], and it has been shown to be involved in major developmental and biological processes, such as spermatogenesis and stem cell maintenance, hind limb formation, hematopoiesis, immune regulation, and oncogenesis [6,7,11,12]. The loss of PLZF has been related to increased proliferation, invasiveness and motility, and resistance to apoptosis in different types of cancer cell lines [8]. Recently, PLZF has been found to be down-regulated in non-small cell carcinoma of the lung [13], malignant mesothelioma [9] and malignant melanoma [8,10]. Overexpression of PLZF in human cervical cell lines and mesothelial cell lines inhibits cell growth by inducing apoptosis [14]. To our knowledge the expression of PLZF has virtually not been studied in prostatic cancer tissue. The aim of this study was to investigate the expression of PLZF in primary as well as metastatic PCa by immunohistochemistry and to correlate the alteration of PLZF expression with PCa grade, aggressiveness as well as metastasis.

## Materials and Methods

### 1. Tissue samples

This study was performed after approval by the institutional review board and in accordance with an assurance filed with and approved by the ethics committee/institutional review board of the University of Rochester and Mount Sinai School of Medicine. This is an exempted immunohistochemical study of archived samples and contains no any identifiable patient information and the need for written informed consent was waived. The prostate gland is a solid fibromuscular organ with ingrowth of glandular epithelia, which it is not readily permeable to formalin fixative. To avoid the possible effect of uneven and suboptimal tissue fixation of the antigen preservation and retrieval as well as on the subsequent immunohistochemical results, biopsy material of primary as well as metastatic prostate cancer was chosen for this study, except for eight cases of radical prostatectomies with pelvic lymph node dissection. All the prostate biopsy specimens and prostatectomies and the majority of metastatic prostate Ca tissue were collected and studied at University of Rochester Medical Center, NY. Twenty percent of the metastatic PCa cases were from Mount Sinai Medical Center, New York. All the cases were from different patients. The specimens were routinely fixed in 10% neutral buffered formalin and paraffin embedded.

There were a total of 83 prostate biopsies, each with a tumor volume ranging from 10 to 100%, among which 13 cases had a total Gleason’s score 6, 57 cases had a score 7, 7 cases has a score 8, and 6 cases had a score 9/10. Benign glandular epithelia were present in 77 of the 83 prostate biopsies and were absent in the remaining 6 biopsies. Forty three cases were metastatic prostate cancer with a tumor volume ranging from 5% to 95%, among which 22 were bone metastases, 11 lymph nodes metastases, 7 liver metastases and 3 lung metastases. Eight paired PCa from prostatectomy specimens and their parallel lymph nodes were also included in the study. The eight primary prostate carcinomas in radical prostatectomies had a total of Gleason’s score ranging from 7 to 9 with a tumor stage pT3a or pT3b. The lymph node metastases ranged from 1 mm to 3mm in greatest dimension. The case distribution are summarized in Table 1.

**Table 1:** Distribution of the cases.

Case type	Number of cases
Prostatic biopsies (total 83)*	
PCa Gleason’s score 6	13
PCa Gleason’s score 7	57
PCa Gleason’s score 8	7
PCa Gleason’s score 9/10	6
PCa metastasis	43
PCa in prostatectomies and parallel pelvic lymph nodes	8

*Benign glands present in 77 of these biopsies. PCa: prostate cancer.

### 2. Immunohistochemistry

Following deparaffinization and rehydration, charged slides with 5-μm thick sections of tissue were treated with 3% hydrogen peroxide (H_2_O_2_) to eliminate endogenous peroxidase activity, then processed for antigen retrieval with 10-mM citrate buffer pH 6.0 using a pressure cooker (Pascal; Dako Cytomation, Glostrup, Denmark) for 1 minute at 125°C, followed by slow cooling. The rest of the procedure was done in a DAKO automated instrument. All sections were rinsed with phosphate-buffered saline (137 mM NaCl, 2.7 mM potassium chloride, 4.2 mM sodium phosphate, and 1.5 mM potassium phosphate) and reacted with mouse anti-PLZF antibody (D-9; sc-28319, Santa Cruz Biotechnology, Santa Cruz, CA) for 1.5 hours at 1:500 dilution in phosphate-buffered saline containing 1% bovine serum albumin (BSA) and 5% normal goat serum at room temperature. The sections were then incubated for 20 minutes with EnVision+ System horseradish peroxidase-labeled polymer conjugated with biotinylated anti-mouse secondary antibody and 3,3'-diaminobenzidine substrate. All slides were counterstained with hematoxylin, dehydrated, and cover slipped.

### 3. Analysis of immunohistochemical staining

Prostate adenocarcinoma, stroma as well as luminal cells and basal cells of benign prostate glands were analyzed for PLZF immunoreactivity in a semiquantitative way. Only nuclear staining of PLZF was considered positive. Based on the extent and intensity of the immunoreactivity, the staining was graded as—(negative/absent), +1 (weak), +2 (moderate), or +3 (strong/intense). Each individual Gleason’s pattern was separately evaluated for their nuclear labeling with PLZF for tumor in the prostate, and for simplicity, Gleason’s pattern 4 (G4) and Gleason’s pattern 5 (G5) were combined at analysis. Gleason’s score was not used for metastatic PCa. The strong lunimal cell staining and weak or non-reactive basal cells (see results section for detail) were used as positive and negative control, respectively.

### 4. Statistical analysis

The Jockheere-Terpstra Test [15] was used to evaluate the trend between protein expression score and cancer type. The test determined if the expected ranks for the protein expression scores and cancer types changed monotonically. Following the test of trend, pairwise comparisons were looked at collapsing protein expression into Negative/Weak (Negative and +1) and Moderate/Strong (+2 and +3) and associating this with pairs of cancer types using 2-tailed Fisher exact tests. All analyses were carried out using SAS/STAT software, Version 9.3 of the SAS System (Copyright © July, 2011, SAS Institute Inc) on a Windows 7 platform.

## Results

### 1. Expression of PLZF in benign prostatic glandular epithelia

**Fig 1:**
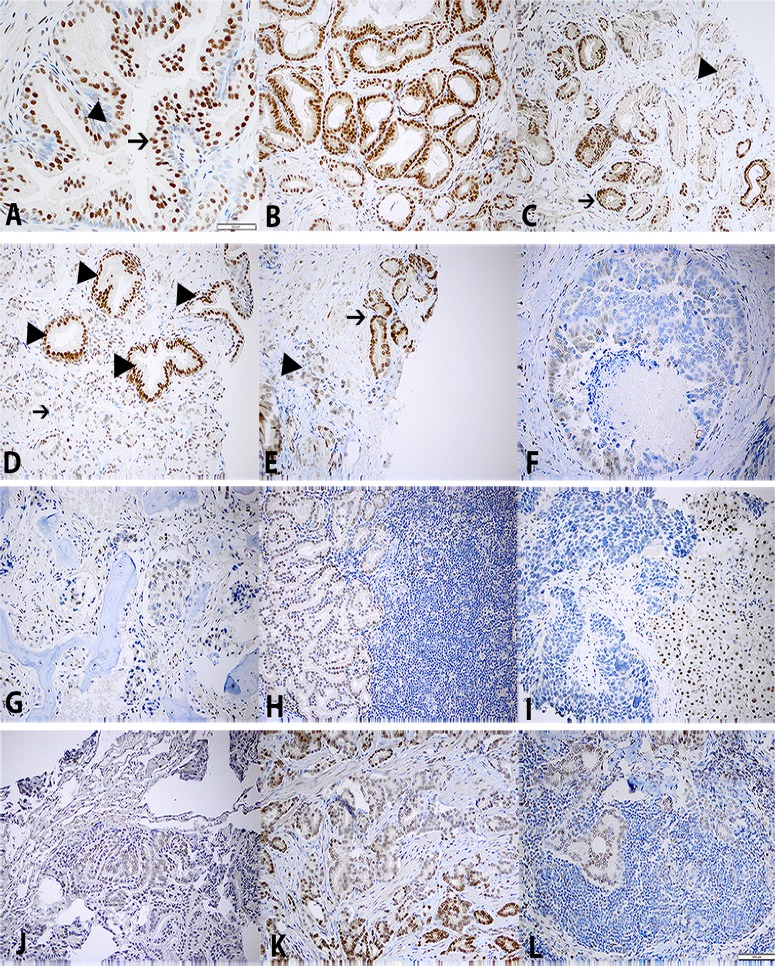
Expression of PLZF detected by immunohistochemistry. A. Benign prostatic glands with strong nuclear imunoreactivity in luminal cells (arrow) and weak or negative labeling in basal cells (arrowhead). B. Strong stain in Gleason’s pattern 3+3 PCa glands. C. Strong stain in G3 PCa glands (arrow), but weak or negative stain in G4 PCa glands (arrowhead). D. Loss of PLZF in G4 glands of Gleason’s pattern 4+4 PCa (arrow). Benign glands with strong stain in luminal cells (arrowhead). E. Strong stain in G3 PCa glands (arrow) and absent stain in G5 PCa glands (arrowhead) of Gleason’s pattern 3+5 PCa. F. Negative stain in Comedo type PCa (G5). G. Negative stain in one representative case of bone metastasis of PCa. H. Negative stain in one representative case of lymph node metastasis of PCa. I. Negative stain in one representative case of liver metastasis. Benign liver also shows nuclear PLZF stain. J. Negative stain in one representative case of lung metastasis. K. PLZF expressed in G3 but not G4 glands of primary PCa from a prostatectomy. L. The parallel lymph node metastasis shows similar morphology and negative PLZF stain to the G4 PCa glands of the primary G4 PCa glands in K.

Among the 83 prostate biopsies, seventy seven contained benign prostate glandular epithelia. PLZF was moderately or strongly expressed in the nuclei of the luminal cells of benign prostatic glands, however, weak or no expression was detected in either the nuclei or cytoplasm of the basal cells or stroma cells in all the 77 cases (Fig. 1A).

### 2. Expression of PLZF in primary prostate cancer of prostate biopsy

Among the primary PCa in the prostate biopsies, there were a total of 70 individual Gleason pattern 3 and 70 individual Gleason’s pattern 4/5. Gleason patterns 4 and 5 were analyzed together mainly due to their similar biologic behavior as well as the absence of obvious immunostaining difference among these two patterns. PLZF was expressed differentially in intensity in the primary prostatic adenocarcinoma of Gleason’s patterns 3 and 4/5. Similar to the luminal cells of benign prostatic glands, PLZF was moderately or strongly expressed in all the tumor glands of Gleason’s pattern 3 (Fig. 1B): 45 showed +3 immunoreactivity and 25 showed +2 positivity. In 3 cases, majority of the G3 PCa glands presented with strong PLZF stain, but minority with focal weak or negative PLZF reactivity. In contrast, among the 70 primary PCa of Gleason’s pattern 4/5, 24 (34%) showed +3, 28 (40%) with +2 and 12 (17%) with weak (+1) immunoreactivity. Complete loss of PLZF expression was seen in 6 (9%) of these cases (Fig. 1D-F). There were 5 cases, in which majority of the G4/5 PCa glands presented with weak or negative PLZF stain, but a minority had focal moderate to strong PLZF reactivity. These PCa with weak or lost expression of PLZF accounted for 18/83 (22%) of all the primary PCa cases and 18/70 (26%) of high grade (Gleason 7–10) primary PCa.

### 3. Expression of PLZF in metastatic prostate cancer

Vast majority of the metastatic PCa did not or had only weakly PLZF expression (Fig. 1G-J). Of the 43 metastatic PCa, 29 lacked PLZF expression (15 bone, 8 lymph node, 5 live and 1 lung), 7 showed weak PLZF expression (5 bone, 1 liver and 1 lung), moderate to strong immunoreactivity of PLZF was seen in 7 cases (2 bone, 3 lymph node, 1 liver, 1 lung). Overall, loss or significantly decreased expression of PLZF was seen in 84% of metastatic prostatic adenocarcinoma regardless of the metastatic site.

### 4. Results of statistical analysis

**Fig 2:**
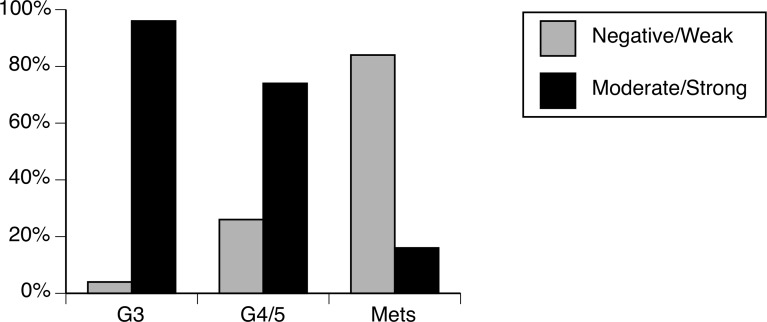
The expression patterns of PLZF significantly different between G3, G4/5 and metastasis and there is significantly decrease in high grade PCa and metastatic PCa (P<0.0001).

The Jockheere-Terpstra Test revealed a negative trend (Z = -9.1568, p<0.0001), indicating that more aggressive metastatic cancer had the lowest protein expression scores, while Gleason scores of 3 or 4/5 had higher protein expression scores. Similarly, highly significant negative associations were found when the collapsed protein score was compared between G3 and G4/5, G3 and metastatic cancer, and G4/5 and metastatic cancer (all p<0.0001, Table 2, Fig. 2).

**Table 2:** Immunostaining results of PLZF expression in benign, primary and metastatic PCa.

Immunostaining scores	Internal benign glands		Primary PCa		Metastatic PCa				
	Luminal cells	Basal cells	G3	G4/5	Bone	LN	Liver	Lung	Total
-	0	62	3*	6	15	8	5	1	29
+1	0	15	0	12**	5	0	1	1	7
+2	29	0	25	28	2	1	1	1	5
+3	48	0	45	24	0	2	0	0	2

G3: Gleason’s pattern 3. G4/5: Gleason’s pattern 4/5.

*. 3 cases of G3 PCa glands with variable PLZF immunostain (-/+1/+2/+3).

**. 5 cases of G4 PCa glands with variable PLZF immunostain (+1/+2/+3). LN: lymph node.

### 5. Expression of PLZF in metastatic PCa of lymph node and its parallel primary PCa

To look into any correlation between the PLZF expression in primary PCa and metastatic PCa, eight paired primary PCa in prostatectomy samples and its corresponding metastatic PCa in lymph nodes were studied for the immunohistochemical expression of PLZF. The results showed that the expression of PLZF in the prostate varied in different grades with moderate to highest expression in low grade (G3) PCa and decreased or lost expression in focus/foci of high grade (G4/5) PCa (Fig. 1K) The focus/foci of primary PCa with decreased or lost expression of PLZF corresponded to the paired metastatic PCa in the lymph node by sharing similar PLZF loss/decrease as well as similar morphologic patterns (Fig. 1L). The immunohistochemical results and pathologic data of the primary and metastatic PCa from the eight radical prostatectomies with pelvic lymph node dissection are summarized in Table 3.

**Table 3:** Immunohistochemical expression of PLZF in primary PCa in the prostatectomies and metastatic PCa in the parallel lymph nodes.

Case	Primary PCa		Parallel lymph node metastasis	Gleason scores	Tumor pTN stage
	G3	G4/5			
1	+1/+2	-/+1/+2	+2	4+3	pTN3aN1
2	+2	-/+1/+2	+1	4+3	pT3bN1
3	+2/+3	-/+1	+1	4+3	pT3aN1
4	NP	+1/+2	+1	5+4	pT3bN1
5	NP	-/+1	-/+1	4+5	pT3aN1
6	+2/+3*	-/+1	-	4+5	pT3bN1
7	+2*	-/+1/+2	+1	4+4	pT3aN1
8	NP	-/+1	-	5+4	pT3bN1

* Minor component of G3 is present in these cases. G3: Gleason’s pattern 3. G4/5: Gleason’s pattern 4/5. NP: Not present.

## Discussion

Although tremendous efforts have been devoted in search of effective prognostic biomarkers that are able to predict the aggressiveness of prostate cancer, little progress has been made toward practical use. By far the most studied biomarker for this purpose is PTEN, a well-known tumor suppressor [16–23]. Genomic deletions of the PTEN locus at 10q23, most commonly identified by FISH, occur in 10% to 70% of prostate cancers depending on the study population examined [16–23]. Some studies have shown the genomic loss of PTEN to be associated with more aggressive behavior, tumor progression and early metastasis, but others did not find such association [23–25]. Given that FISH test is cumbersome and does not detect epigenetic as well as post-transcriptional alteration related gene expression, immunohistochemical tests have recently been used to assess the expression of PTEN in prostate cancer and its relation to tumor aggressiveness. Inconsistent results however have been reported [21, 23, 26–33]. Lack of reliable antibodies against PTEN has been regarded as one the attributing factors. PTEN immunoreactivity has been shown in the nuclei and cytoplasm of epithelial cells as well as in the stroma cells of prostate tissue [28] and there has been no consensus on the immunostaining scoring as well as on the interpretation of the PTEN immunohistochemical results; when correlation to clinicopathologic parameters is performed, cytoplasmic PTEN staining is used by some authors [27, 34], while nuclear reactivity is used by others [23]. In addition, promiscuous immunostaining in the nucleus and cytoplasm of epithelial cells as well as stroma cells may also have made it difficult to distinguish specific from nonspecific reactions. Furthermore, the quality of tissue preservation, a frequently overlooked factor, has been known to affect the immunohistochemical results [35, 36]. In order to correlate the results of PTEN expression to tumor stage, most of the previous studies used prostatectomy samples, including prostatectomy tissue derived TMA (tissue microarray). As aforementioned, prostate gland is mainly a solid fibromuscular organ with a low permeability to fixative; therefore, unless special attention is paid, suboptimal tissue fixation is a common issue for prostatectomy specimen, which subsequently could affect the tissue antigen preservation and might at least partly explain the inconsistent results in some of the immunohistochemical studies of PTEN in PCa. As a result, the clinical value of immunohistochemical PTEN in the assessment of prostate cancer aggressiveness is still under evaluation.

PLZF, a gene modulator similar to PTEN, functions as a transcription repressor. Previous studies of PLZF have been mainly focused on its role in spermatogenesis, stem cell maintenance and promyelocytic leukemia [6,7,11,12]. Our study in prostate demonstrated that, in comparison to the moderate to strong immunohistochemical expression in benign luminal cells of prostate glands and low grade (Gleason’s pattern 3) prostate cancer, expression of PLZF was significantly decreased or lost in vast majority (84%) of metastatic prostate cancer and in 26% of high grade primary PCa. These results indicated that loss of PLZF was an important step in tumor progression and metastasis. A fraction (26%) of primary high grade (G4/5) prostate cancer presented with significant loss of PLZF expression also seemed to fit in the real clinical scenario, in which only a portion of high Gleason PCa progress and develop metastasis along their courses. The 26% of these primary high grade PCa with significant decrease or loss of PLZF expression in our study might therefore actually represent the portion of aggressive PCa that develop metastasis at later time. There were occasionally cases with variable PLZF expression in the same Gleason’ score PCa glands of the same biopsy, which is consistent with the intratumor heterogeneity of PCa.

To further elucidate whether or not the decrease or loss of PLZF expression in primary PCa correlated to that of the metastatic PCa, we studied the immunohistochemical expression pattern of PLZF in both primary PCa in the prostate and the paired metastatic PCa in dissected pelvic lymph nodes of the same patient. Our results showed the changes of PLZF expression as well as the morphology in the focus/foci of primary high grade PCa in the prostate was correlated to that of the pelvic lymph node metastatic PCa. This finding strengthened the association of PLZF loss with PCa progression and implicated a prognostic value of PLZF loss in primary PCa in predicating its metastatic potential. In the paired prostatectomy and lymph node dissection, the variable PLZF expression (-/+1/+2/+3) in the primary G4/5 PCa glands may reflect the variability of antigen presentation due to suboptimal fixation of the prostatectomy specimens, nevertheless, proportionally, the PLZF in the foci of G4/5 PCa are less expressed than the foci of G3 PCa.

As demonstrated in this study, PLZF was almost exclusively expressed in the nuclei of luminal cells and weak or absent in the basal cells and stromal cells of the prostate. This clean background not only made it easy to interpret the immunostaining results, but also served as excellent positive and negative controls. These were the obvious advantages of PLZF over PTEN. In addition, compared to the loss of PTEN in 10–70% of PCa [16–23], the sensitivity of PLZF loss for metastatic PCa was 84% in our study, while the specificity of PLZF loss in predicting no metastasis from low grade (Gleason 3) was 70/70 (100%) or at a minimum, 67/70 (94%) when taking into account minor focal loss. Precisely how many of the high grade (G4/5) PCa will fail to develop metastasis is nearly impossible to ascertain in human cases; nevertheless, if one assumes that the entire group of 52 (74%) primary G4/5 PCa cases that did not display PLZF loss lacks metastatic potential, the specificity of PLZF in predicating metastasis would be much higher than the reported 50% for PTEN [37].

We have examined the expression of PLZF using Oncomine (Life Technologies) as well as the underlying GEO data in five prostate cancer datasets stratified by Gleason score. No apparent association is evident, neither when examining the stratified expression box plots (S1 Fig.), nor the underlying data when arrayed as waterfall plots (S2 Fig.). Similarly, when examining five publicly available datasets at cBioPortal [38, 39], no strong association between Gleason score (S3 Fig., top three sets) or T-stage (S3 Fig., bottom two sets) and homozygous deletion, amplification, nor mutation of the gene encoding PLZF (ZBTB16) could be discerned. In addition, no apparent association was seen between putative copy number alterations and PLZF mRNA expression in these datasets accessed through cBioportal (data not shown). The discrepancy between the genomic and expression data and our immunohistochemical staining results suggests an involvement of epigenetic and/or posttranscriptional alterations in aggressive prostate cancer and supports a more informative role for immunohistochemical proteomic analysis of the end product of a gene in elucidating its function/biological consequence in the understanding and prediction of tumor behavior.

Although the exact role of PLZF in prostate epithelium is unknown, The constitutive expression of PLZF in the luminal cells and absent in basal cells of benign prostatic glands is reminiscent of androgen receptor, and strongly infers an association of PLZF with androgen receptor. In fact, in an *in vitro* study of PCa cell lines, PLZF is found to be a prostatic androgen-responsive gene [40]. PLZF is upregulated by androgen in the androgen-responsive cell line LNCaP, but not in the androgen receptor negative cell line DU145 [41]. Overexpression of PLZF in the LNCaP cell line inhibits cell proliferation both in the absence and presence of androgens [40]. Endogenous PLZF expression is absent in androgen-independent PCa cell line DU145 and its expression can be restored by ectopic expression of the androgen receptor, but this is not true in androgen- dependent cell line LNCaP [41, 42]. The results of these in vitro cell line studies support a relationship between PLZF and androgen receptor activity, and indicate that the balance between the pro-proliferative androgen receptor and inhibitory action of PLZF may play a crucial role in the maintenance of the homeostasis of benign prostate epithelium. Loss of PLZF activity in PCa may result in unchecked androgen receptor activity, which would lead to proliferative growth. While the underlying mechanisms of PLZF loss and related tumor progression as well as its role in PCa androgen independence have yet to be investigated, these data motivated us to examine the PLZF expression in prostate cancers with various grades (Gleason’s scores) as well as metastatic prostate cancers.

In summary, our study demonstrates that the immunohistochemical loss of PLZF is a sensitive and specific biomarker in detecting aggressive and metastatic PCa. Nevertheless we would also like to emphasize that the scope of this study is limited and a larger scale study to validate the results is needed. If further validated, due to the simplicity and cost-effectiveness, immunohistochemical testing of PLZF expression in PCa will have a significant impact and be of clinical utility in the management and personalized care of PCa patients in daily practice. PLZF immunohistochemistry may also be combined with PTEN to provide increased predictive power for progressive PCa. Prospectively restoring PLZF in these aggressive PCa may also have potential therapeutic values by reversing their courses.

## Supporting Information

S1 Fig No alterations in PLZF mRNA in prostate cancer. PLZF (ZBTB16) expression was examined in five prostate cancer datasets stratified by Gleason score, available in Oncomine. Only datasets with 10 or more cases of Gleason score 8 and 9 were analyzed. Box plots depict mean expression, stratified by Gleason score. Stratification in columns by Gleason score indicated in legend below (with number of cases indicated parenthetically). Note that the appropriate comparison is between Gleason score 7 and lower versus Gleason score 8 and or 9, and not the first column for sets 1–4, which depicts samples for which a score was not available.
(TIFF)

S2 Fig No increased frequency of cases of reduced PLZF mRNA expression in high grade prostate cancer. PLZF (ZBTB16) expression was examined in five prostate cancer datasets stratified by Gleason score, available in Oncomine. Only datasets with 10 or more cases of Gleason score 8 and 9 were analyzed. Waterfall plots portray the underlying individual data analyzed in S1 Fig. and depict mean expression, stratified by Gleason score. Stratification in columns by Gleason score indicated in legend below (with number of cases indicated parenthetically). Note that the appropriate comparison is between Gleason score 7 and lower versus Gleason score 8 and or 9, and not the first column for sets 1–4, which depicts samples for which a score was not available.
(TIFF)

S3 Fig No PLZF gene mutations or alterations in prostate cancer. Disruptions of the gene encoding PLZF (ZBTB16) were examined in the indicated five datasets available at cBioportal. Alterations are indicated in plots below study name: homozyogous deletion (blue bars); amplification (red bars); mutation (green bars). Sum of all detected alterations was 0%, 4%, 1%, 2%, and 4% (top to bottom) in the studies depicted.
(TIFF)
