# Supplementary figures and images for: Loss of PLZF Expression in Prostate Cancer by Immunohistochemistry Correlates with Tumor Aggressiveness and Metastasis
Source: PLoS One. 2015 Mar 25;10(3):e0121318. doi: 10.1371/journal.pone.0121318 (PMC4373907; doi:10.1371/journal.pone.0121318)

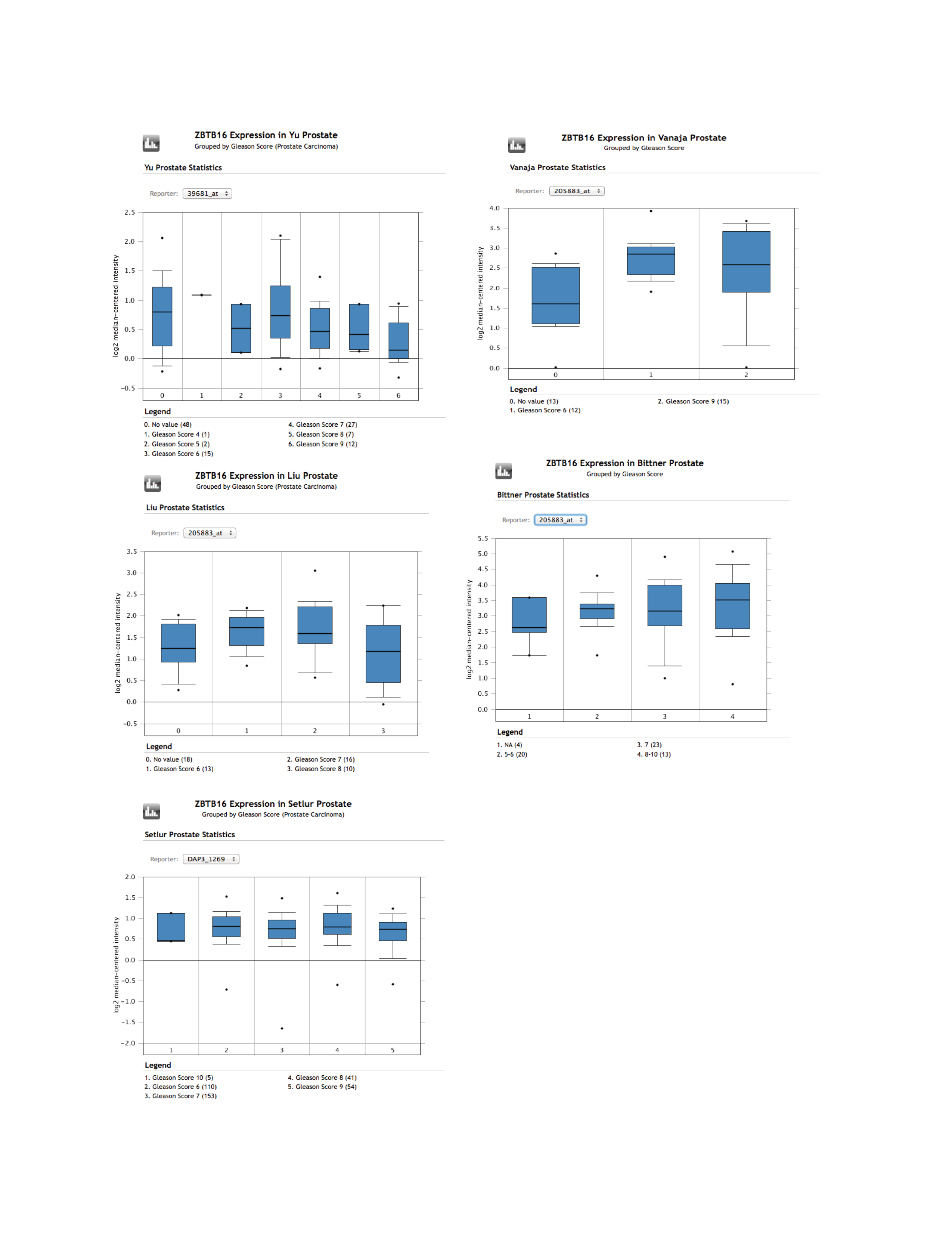

Supplement: S1 Fig — PLZF (ZBTB16) expression was examined in five prostate cancer datasets stratified by Gleason score, available in Oncomine. Only datasets with 10 or more cases of Gleason score 8 and 9 were analyzed. Box plots depict mean expression, stratified by Gleason score. Stratification in columns by Gleason score indicated in legend below (with number of cases indicated parenthetically). Note that the appropriate comparison is between Gleason score 7 and lower versus Gleason score 8 and or 9, and not the first column for sets 1–4, which depicts samples for which a score was not available. (TIFF) [file pone.0121318.s001.tiff]

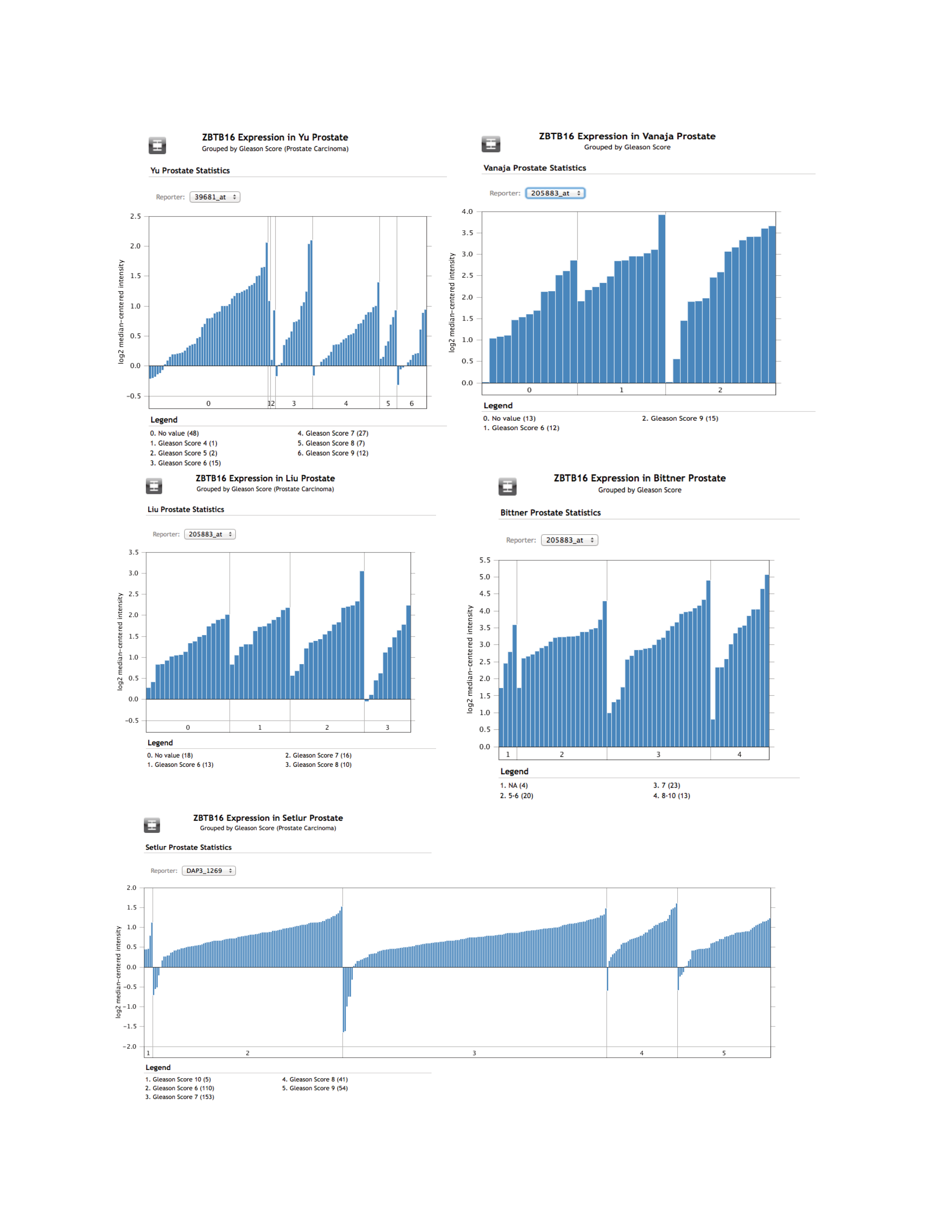

Supplement: S2 Fig — PLZF (ZBTB16) expression was examined in five prostate cancer datasets stratified by Gleason score, available in Oncomine. Only datasets with 10 or more cases of Gleason score 8 and 9 were analyzed. Waterfall plots portray the underlying individual data analyzed in S1 Fig. and depict mean expression, stratified by Gleason score. Stratification in columns by Gleason score indicated in legend below (with number of cases indicated parenthetically). Note that the appropriate comparison is between Gleason score 7 and lower versus Gleason score 8 and or 9, and not the first column for sets 1–4, which depicts samples for which a score was not available. (TIFF) [file pone.0121318.s002.tiff]

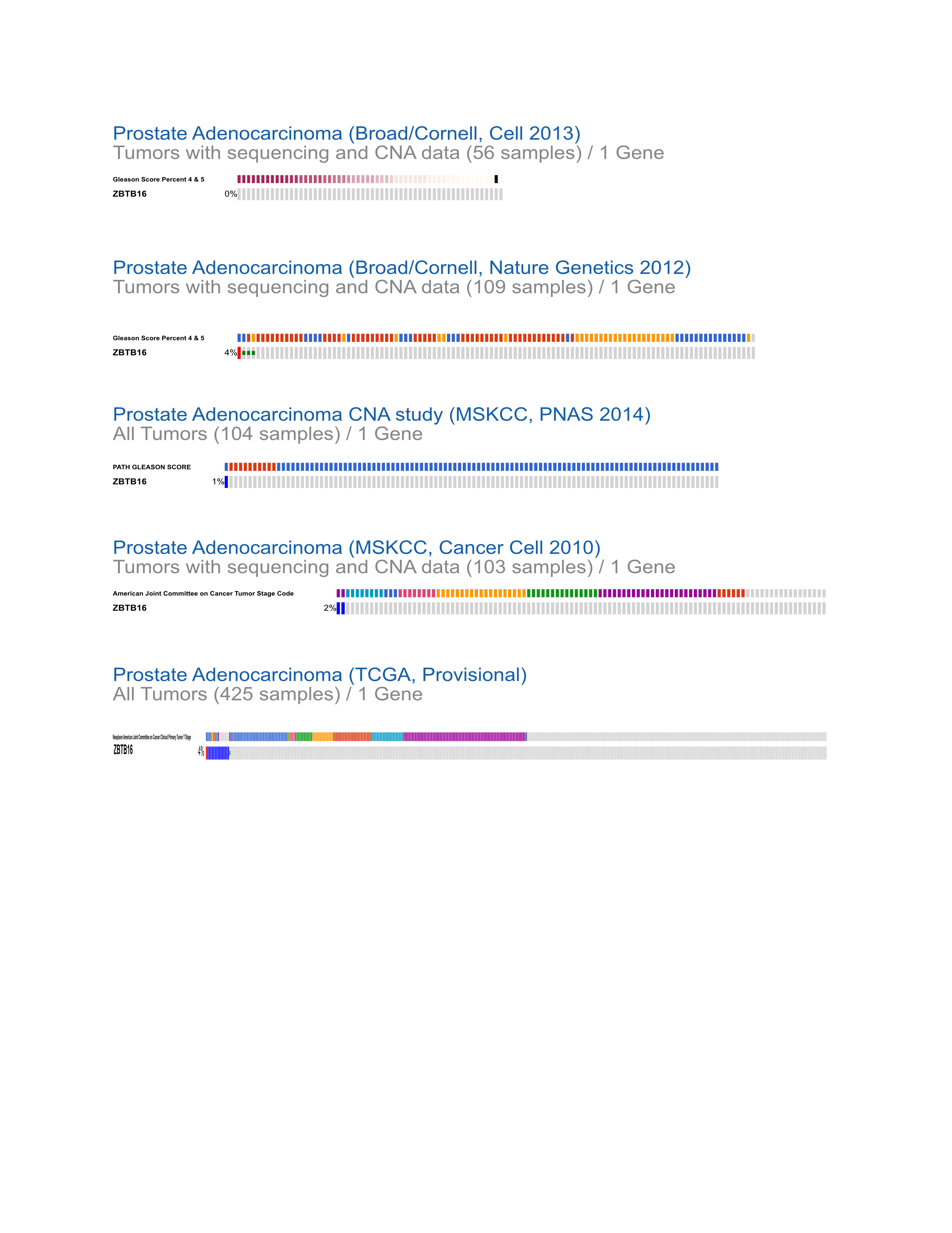

Supplement: S3 Fig — Disruptions of the gene encoding PLZF (ZBTB16) were examined in the indicated five datasets available at cBioportal. Alterations are indicated in plots below study name: homozyogous deletion (blue bars); amplification (red bars); mutation (green bars). Sum of all detected alterations was 0%, 4%, 1%, 2%, and 4% (top to bottom) in the studies depicted. (TIFF) [file pone.0121318.s003.tiff]
